# Chronic exposure to biomass ambient particulate matter triggers alveolar macrophage polarization and activation in the rat lung

**DOI:** 10.1111/jcmm.17169

**Published:** 2022-01-06

**Authors:** Shenlin Wang, Yuhua Chen, Wei Hong, Bing Li, Yumin Zhou, Pixin Ran

**Affiliations:** ^1^ State Key Laboratory of Respiratory Diseases National Clinical Research Center for Respiratory Diseases Guangzhou Institute of Respiratory Health The First Affiliated Hospital of Guangzhou Medical University Guangzhou Guangdong China; ^2^ Department of Respiratory Medicine Ningxia Hui Autonomous Region People's Hospital The First Affiliated Hospital of Northwest University for Nationalities Yinchuan Ningxia China; ^3^ GMU‐GIBH Joint School of Life Sciences Guangzhou Medical University Guangzhou Guangdong China

**Keywords:** alveolar macrophage, ambient particulate matter, biomass fuel, inflammation, remodelling

## Abstract

The role of alveolar macrophages (AMs) in chronic obstructive pulmonary disease is unclear. We characterized the function of AMs in rats chronically exposed to biomass fuel smoke (BMF) and studied the signal pathways that regulate AMs polarization. One hundred and eighty male Sprague‐Dawley rats were divided into BMF group and clean air control (CON) group. After BMF smoke exposure for 4 days, 1 month and 6 months, the cytokine secretion and function of AMs were determined by flow cytometry, quantitative polymerase chain reaction, Western blotting and immunofluorescence. Bone marrow‐derived macrophages were cultured and exposed to particulate matter (PM) from the smoke. Exposure initially promoted pro‐inflammatory factors, but pro‐inflammatory macrophages shared features of anti‐inflammatory macrophages. Consistent with IL‐4 upregulated in bronchoalveolar lavage fluid, p‐Stat6 and peroxisome proliferator‐activated receptor γ (PPARγ) in AMs elevated at 4 days of exposure. After 6 months of exposure, CD206, TGF‐β1 and p‐Smad3 were significantly higher than the control groups. PPARγ reversed the M1 phenotype induced by PM in vitro and drove the macrophages into the M2 phenotype. Altogether, the study demonstrates the dynamic phenotype and functional changes in AMs during exposure to BMF smoke.

## 1 INTRODUCTION

Chronic obstructive pulmonary disease (COPD) has high disability and fatality rates and imposes great economic and social burdens. The prevalence of COPD among individuals over 40 years old is estimated at 13.7% in China.^1^ Factors that influence COPD development and progression are extremely complex, and smoking is not the only factor. Almost three billion people worldwide use wood as the main source of energy for cooking, heating and other household needs. Indoor biomass smoke exposure increases the risk of COPD, and accounts for 50% of deaths in patients with COPD in developing countries,^2^ and increases in COPD hospitalization.^3^, ^4^, ^5^, ^6^, ^7^ Alterations in cooking fuels and kitchen ventilation can halt the associated decline in forced expiratory volume in 1 s (FEV_1.0_).^8^ Although the pathologic features of COPD induced by biomass ambient particulate matter (PM) are less severe than those of COPD induced by smoking,^9^ their hazards should not be ignored. However, the molecular pathway of pathological damage caused by biomass ambient particulate matter in vivo and in vitro remains less understood.

COPD is characteristic of a mixture of small‐airway disease and parenchymal destruction. Most research has focused on airway inflammation, and links between changes to host defence and lung immunity. Alveolar macrophages (AMs) form the first line of immune defence in lung tissue and maintain lung local immune homeostasis. The pattern recognition receptors of AMs identify invading pathogens and initiate the inflammatory response. When the dangerous substances are cleared, macrophages can secrete anti‐inflammatory mediators and growth factors to promote the elimination of inflammation and tissue repair. The immune phenotype and function of AMs are greatly affected by the local microenvironment of the alveolar lumen.

Macrophages can be classified into classical activated M1 macrophages and alternative activated M2 macrophages.^10^ When stimulated by type 1 T helper (Th1) cytokines such as interferon ‐γ (IFN‐γ) and toll‐like receptor signalling, M1 is activated.^11^ M1 expresses CD86, secretes interleukin (IL)1 and tumour necrosis factor (TNF)α and can kill pathogenic microorganisms.^12^ M1 also secretes IL‐6, IL‐12 and IL‐23 to promote the differentiation of Th1 and type 17 T helper (Th17) cells and promote the inflammatory response.^13^ The signal pathway molecules include Stat1, nuclear factor kappa B (NFκB) and mitogen‐activated protein kinases (MAPKs). The antigen presentation capacity of AMs is very weak, inhibits response to harmless pathogens and reduces the release of inflammatory cytokines.^14^, ^15^ When stimulated by Th2 cytokines such as IL‐4 and IL‐13, M2 activates and expresses CD206, CD163, transforming growth factor (TGF)β, tyrosine‐protein kinase, arginase 1, Stat6 and Stat3, which are involved in tissue repair and anti‐inflammation.^13^, ^16^, ^17^ M2 also secretes epidermal growth factor (EGF) and vascular endothelial growth factor (VEGF) to promote tissue repair.^17^ M2 is divided into several subgroups: the traditional M2 phenotype is known as M2a, and macrophages stimulated with IL‐10 are termed M2c. M2a attenuates inflammation and stimulates tissue repair, while M2c promotes tissue remodelling. With stimulation by IL‐4, nuclear receptor peroxisome proliferator‐activated receptor (PPAR)γ is upregulated in macrophages, skewing macrophages towards the M2a phenotype.^18^, ^19^, ^20^ M2‐derived TGF‐β contributes to fibrosis pathology by upregulation of α‐smooth muscle actin(α‐SMA) via the Smad pathway.

M1 macrophages predominate in non‐allergic inflammation model, while the M2 phenotype predominates in allergic asthma.^21^ COPD patients have higher levels of AMs and inflammatory mediators that contribute to the pathology of the disease.^22^, ^23^ In contrast, AMs in the bronchoalveolar lavage fluid (BALF) of COPD patients have been reported to express M2 markers.^24^ Both isoforms of nitric oxide synthase (iNOS) and CD206 were shown to expressed by macrophages in the lungs of non‐COPD smokers and COPD patients, indicating that macrophages in the lungs were polarized bidirectionally.^25^ The function of AMs in COPD is unclear. Diversity and plasticity are characteristics of macrophages in vivo.^26^ In order to maintain the homeostasis of the body, immunity constantly undergoes dynamic changes during the development and progression of the disease, as do AMs. To our knowledge, there are no published studies on dynamic changes in AMs in COPD.

In the present study, we exposed rats to biomass ambient particulate matter to study phenotypic changes and immune function of AMs in vivo, and investigate the transcriptional factors involved in activation of AMs.

## 2 METHODS

### 2.1 Animals

One hundred and eighty male Sprague‐Dawley rats (170–200 g, 6–8 weeks old) were housed in the laboratory animal centre of Guangzhou Medical University under barrier conditions. The rats were randomly divided into a biomass fuel (BMF) group and clean air control (CON) group. The experimental protocol and animal care were in compliance with the guiding principles for the care and use of laboratory animals recommended by the Chinese Association for Laboratory Animal Science Policy. Guangzhou Medical University Animal Research Ethics Board approved all experiments.

### 2.2 BMF smoke exposure system

Rats were exposed to smoke produced by smouldering China fir saw‐dust (2 g per heating panel per session) for 2 h periods, 4 days and 5 days per week for 1 month and 6 months. The BMF smoke exposure system primarily consisted of a wood‐burning unit and a whole‐body exposure unit. The size of the animal exposure chamber was 265 × 205 × 140 mm (length by width by height). BMF smoke was generated by eight heating panels (500 w), which were connected in series in a wood‐burning chamber. Each heating panel functioned for 20 min before the next heating panel was activated. BMF smoke was set into the animal exposure chamber through a pump (15 L/min), while exhaust gas was pumped away by a negative pressure pump at a rate of 15 L/min, and continuous fresh air was poured into wood‐burning chamber. Besides, there were two sampling ports to monitor various characteristics of PM and gas in exposure chamber.

### 2.3 Particulate matter (PM) collection and extraction

PM was collected from the burning of China fir during high‐temperature combustion with a moderate air supply between April 23 and May 6 of 2015 in accordance with a procedure described previously.^27^ A high‐volume sampler (TE‐6070; Tisch Environmental) equipped with a fine particulate matter selective‐inlet head(1.13 m^3^/min) was used to collect particles. Exposed filters were soaked in water for 10 min and then in dichloromethane for 4 h. The extracted solution was lyophilized and mixed. The weight of PM was defined as the increase in the weight of each filter. The PM sample was dissolved in dimethyl sulfoxide (DMSO) to a volume of 100 mg/ml, and then diluted with culture medium to yield a concentration of <0.01% DMSO.

### 2.4 Sample preparation and isolation of AMs

Rats were sacrificed after 4 days, 1 month and 6 months of exposure. BALF was obtained by instilling the lungs sequentially with 8 ml ice‐cold phosphate‐buffered saline (PBS) four times. BALF was centrifuged to obtain cells and supernatants. The cells were suspended with 1 ml PBS and counted with a cell counter (Millipore Scepter2.0; MilliporeSigma). Cells were then plated onto six‐well plates (100 × 10^4^ cells/well; Corning) at 37℃ in a humidified atmosphere of 5% CO_2_. After 12 h of incubation, non‐adherent cells were removed and adherent cells were used in the quantitative polymerase chain reaction (qPCR) analyses of mRNA.

### 2.5 Culture and stimulation of bone marrow‐derived macrophages (BMDMs)

Bone marrow cells were obtained from the femur and tibia bones and incubated in RPMI‐1640 medium supplemented with 10% heated‐inactivated foetal bovine serum and recombinant rat granulocyte‐macrophage colony‐stimulating factor (GM‐CSF) (10 ng/ml; PeproTech) for 7 days as described.^28^ PM was used at concentrations of 0–45 μg/ml. Rosiglitazone (ROG) (1 μM; Sigma‐Aldrich), IL‐4 (50 ng/ml; PeproTech) and TGF‐β1 (10 ng/ml; PeproTech) were added 1 h before PM treatment.

### 2.6 CRISPR/Cas9‐mediated PPARγ gene knockout in BMDMs

LentiCrisp/Cas9 system was used to knock out PPARγ from bone marrow‐derived macrophages (BMDMs) with the GeCKO LentiCrisp Resource Tool. Guide RNAs(gRNA) were designed using MIT’s online webpage (http://crispr.mit.edu/).

The short guide RNA sequences for PPARγ were as follows: 5′‐CCTGTGGAGGTCCCATAATA‐3′ and 5′‐TAATACCTTACCTAGTATCG‐3′. The vector included the Cas9 gene. Lentivirus to knock out PPARγ was constructed by Cyagen Biosciences.

### 2.7 Gene expression

Total RNA was extracted and reverse‐transcribed by using the PrimeScript RT reagent kit with gDNA Eraser (Takara Bio). qPCR was performed by using TB Green Premix Ex Taq (Takara Bio). The reactions were run on a CFX real‐time detection system (Bio‐Rad Laboratories). Table S1 lists the qPCR primer sequences.

### 2.8 Flow cytometry

Total BALF cells were incubated with fixable viability dye eFluor450 (Thermo Fisher Scientific) for 30 min at 4℃. After blocking with Fc receptors (BD Biosciences), cells were incubated with CD206‐PE (Polyclonal, IgG; R&D Systems), PE Isotype Control (IgG; R&D Systems), CD86‐PE (clone 24F, IgG1,κ; BD Biosciences) and PE Isotype Control (clone 24F, IgG1,κ; BD Biosciences) for 50 min at 4℃, respectively. After fixation and permeabilization, cells were incubated with CD68‐Alexa Fluro 647 (clone ED1, IgG1; Bio‐Rad Laboratories) and Alexa Fluor 647 Isotype Control (clone ED1, IgG1; Bio‐Rad Laboratories) for 50 min at 4℃. Flow cytometry was conducted on a Beckman Coulter CytoFLEX instrument (Brea).

### 2.9 Cytokine assessment

BALF supernatants were used to quantitatively measure 27 rat cytokines/chemokines using a magnetic bead panel (RECYMAG65K27PMX; MilliporeSigma).

### 2.10 Western blotting

Lung tissue was lysed by using RIPA buffer (Thermo Fisher Scientific). Equal amounts of proteins were separated by 10% sodium dodecyl sulphate‐polyacrylamide gel electrophoresis and transferred to polyvinylidene fluoride membranes (Bio‐Rad Laboratories). The membrane was blocked with 5% bovine serum albumin (Sigma‐Aldrich) for 90 min at room temperature and incubated with a primary antibody for 14 h at 4℃. The primary antibodies included anti–PPAR‐γ (Abcam), anti‐TGF‐β1 (ProteinTech), anti‐phosphor‐Smad3 (Cell Signaling Technology), anti‐Smad3 (Cell Signaling Technology), anti‐phosphor‐Stat6 (Cell Signaling Technology), anti‐Stat6 (Santa Cruz Biotechnology), anti‐phosphor‐Stat3 (Cell Signaling Technology), anti‐Stat3 (Santa Cruz Biotechnology), anti‐β‐tubulin (ProteinTech), anti‐NFκB P65 (Abcam), anti‐IκBα (Abcam), anti‐IL‐1β (Abcam), anti‐phosphor‐NFκB P65 (Cell Signaling Technology), anti‐phosphor‐IκBα (Cell Signaling Technology) and anti‐GAPDH (Abcam). And then, membranes were incubated with anti‐rabbit/mouse IgG (H+L) (Abcam) for 1 h at room temperature. Chemiluminescence measurement was performed by using Amersham Imager 680 (GE Healthcare Life Science).

### 2.11 Histological staining

The lavaged lung (left) was then inflated with 4% formaldehyde and maintained at a pressure of 25 cmH_2_O to keep for histological assessment. Sections (5 μm) were stained with haematoxylin and eosin (HE) to assess morphological changes.

### 2.12 Immunofluorescent staining

Formaldehyde‐fixed lung sections (5 μm) were dewaxed in xylene and rehydrated in ethanol/water. After antigen repair solution, 0.05% Triton X‐100 permeabilization and blocking with 10% goat serum, the sections were incubated with primary antibodies, including rabbit anti‐PPAR‐γ (Abcam), rabbit anti–phosphor‐Stat6 (Cell Signaling Technology), rabbit anti‐TGF‐β1 (ProteinTech), rabbit anti‐phosphor‐Smad3 (Cell Signaling Technology), rabbit anti‐phosphor‐NFκB P65 (Cell Signaling Technology) and rabbit/mouse anti‐CD68 (Abcam) at 4℃ overnight, respectively. Sections were washed three times and incubated with Alexa Fluor 488‐conjugated goat anti‐rabbit IgG (Abcam), Alexa Fluor 647‐conjugated donkey anti‐mouse IgG (Abcam) and CoralLite 594‐conjugated goat anti‐rabbit IgG (ProteinTech) at 37℃ for 1 h, and then labelled with DAPI for 5minutes. Immunofluorescence was measured by a confocal microscope (Carl Zeiss).

### 2.13 Statistical analysis

Statistical analyses were performed using IBM SPSS 22.0 (Armonk, NY, USA), and data were expressed as mean ± standard deviation (SD). Two‐group comparisons were conducted using an unpaired *t* test. Comparisons of more than two groups were performed using one‐way ANOVA test. The Mann‐Whitney *U* test was used to compare relative mRNA expression and CD206 mean fluorescence intensity (MFI) between experimental groups. *p *< 0.05 was considered significant.

## 3 RESULTS

### 3.1 Determination of particle size distributions and gas concentrations in the exposure chamber

To measure the particle size distributions in suspension and gas concentrations as benchmarks of quality control parameters of the exposure system, we used a DustTrakⅡ aerosol detector (TSI) and a Test340 portable gas analyzer (Testo). The concentrations of particulate matter with diameters of 1, 2.5 and 10 μm (PM_1_, PM_2.5_ and PM_10_) were 27.77 ± 8.66, 28.07 ± 8.84 and 28.23 ± 8.86 mg/m^3^ in the BMF exposure room, respectively (Table S2). The carbon monoxide (CO) concentration was maintained at 55.16 ± 13.77 ppm, and nitric oxide (NO) and sulphur dioxide (SO_2_) were not detected.

### 3.2 BMF smoke‐induced lung morphological changes and AMs infiltration

**FIGURE 1:**
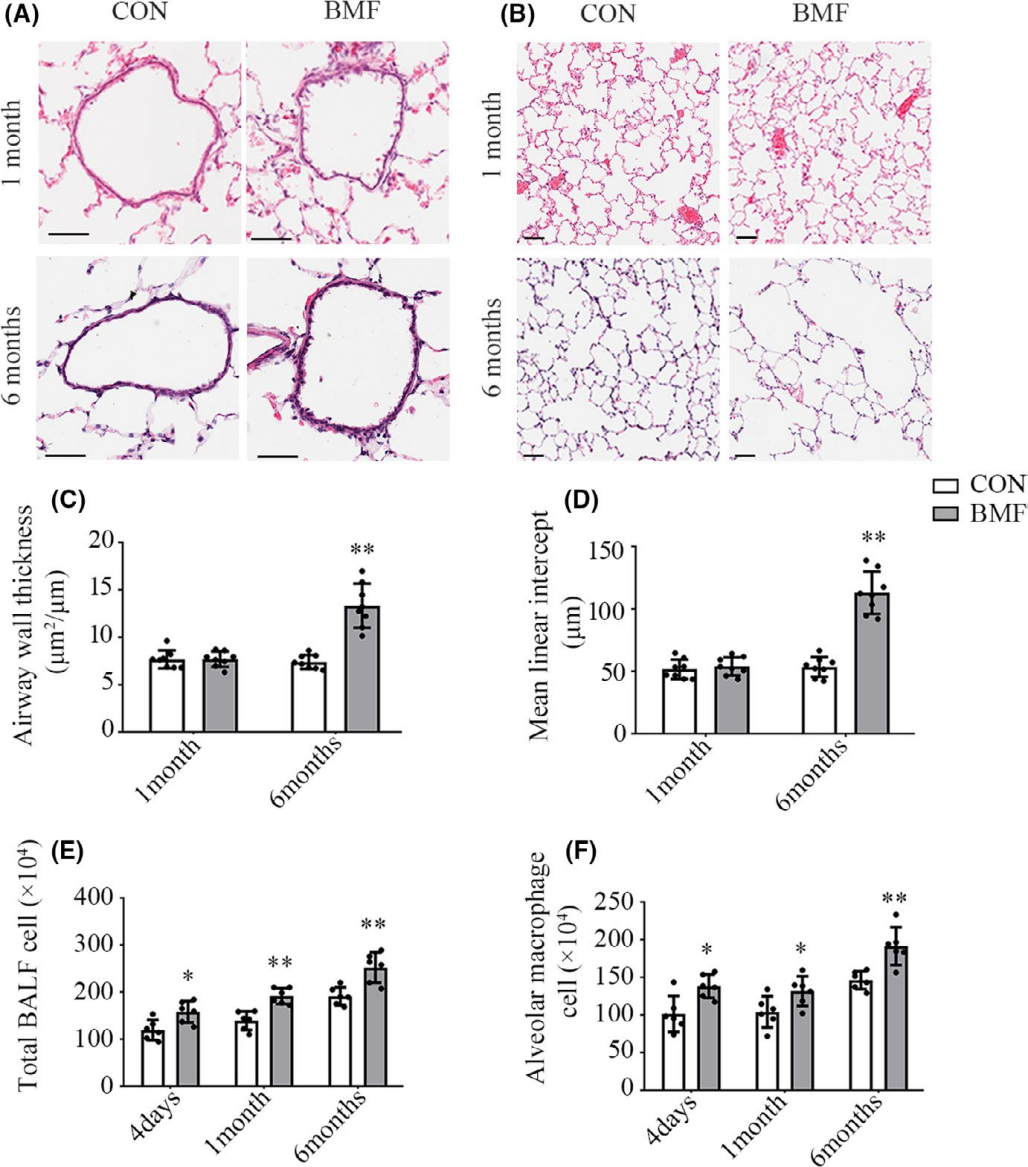
Lung morphological changes and AMs infiltration into BALF following exposure to smoke from BMF. Scale bar: 50 μm. A, C: Small‐airway wall stained with HE and statistical analysis of small‐airway wall thickness. B, D: Lung tissue stained with HE and statistical analysis of mean linear intercept (MLI). E: Comparison of the total number of cells in BALF between BMF and CON groups. F: Comparison of AMs in BALF between BMF and CON groups. Data in C, D, E, and F represent the mean ± SD of a minimum number of six rats per group. *p < 0.05, **p < 0.01, significantly different from CON groups. AMs, alveolar macrophages; BALF, bronchoalveolar lavage fluid; BMF, biomass fuel smoke; CON, control

We assessed morphometric changes in the lungs as markers of emphysema. Alveolar enlargement was calculated as the mean linear intercept (MLI), and the bronchial wall thickness was quantified by wall thickness, calculated as the total bronchial area minus the lumen area, divided by total bronchial area. BMF smoke exposure‐induced emphysematous changes and airway remodelling (Figure 1A–D). Long‐term BMF smoke exposure damaged the lung parenchyma and airway wall, resulting in alveolar enlargement and distal airway remodelling. Histological analysis demonstrated that the airway wall thickness increased (*p *< 0.01), and the mean linear intercept decreased (*p *< 0.01) after a 6‐month exposure. No changes were observed after 1 month of exposure (*p *= 0.366 and 0.557). The number of cells in BALF increased after 4 days, 1 month and 6 months of BMF smoke exposure (Figure 1E, *p* = 0.013, 0.001 and 0.003, respectively). AMs were labelled with the pan macrophage surface marker CD68 and defined as the CD68^+^ subpopulation with the purity displayed as a percentage of parent population gated on FSC‐A/SSC‐A. The numbers of infiltrated AMs increased after 4 days, 1 month and 6 months of BMF smoke exposure (Figure 1F, *p* = 0.01, 0.04 and 0.003, respectively), and peaking at 6 months.

### 3.3 BMF smoke exposure‐induced BALF cytokine expression

**FIGURE 2:**
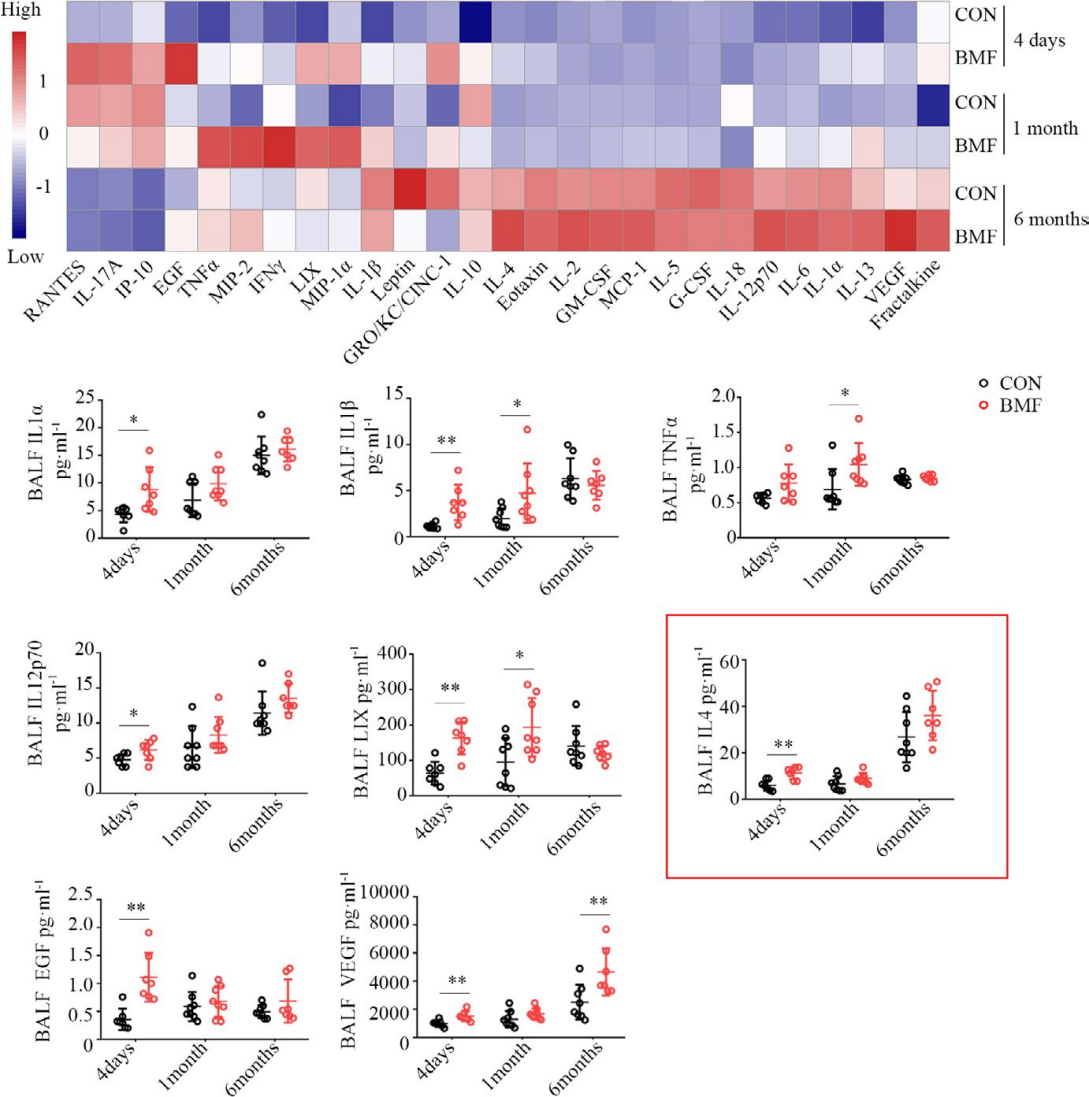
Smoke from BMF‐induced cytokine expression in BALF. Analysis of 27 cytokine showed the levels of IL‐1α, IL‐1β, IL‐12p70, LIX, TNFα, IL‐4, EGF and VEGF. Data represent the mean ± SD of a minimum number of six rats per group. *p < 0.05, ** *p* < 0.01, significantly different from CON groups. BALF, bronchoalveolar lavage fluid; BMF, biomass fuel smoke; CON, control; EGF, epidermal growth factor; VEGF, vascular endothelial growth factor

To investigate how BMF exposure influences pulmonary inflammation, which may affect the M1/M2 phenotype, 27 cytokines/chemokines multiplex tests were performed (Figure 2). We measured granulocyte colony‐stimulating factor (G‐CSF), eotaxin, GM‐CSF, IL‐1α, leptin, MIP‐1α, IL‐4, IL‐1β, IL‐2, IL‐6, EGF, IL‐13, IL‐10, IL‐12p70, IFNγ, IL‐5, IL‐17A, IL‐18, MCP‐1, IP‐10, GRO/KC/CINC‐1, VEGF, fractalkine, LIX, MIP‐2, TNFα and RANTES protein levels by using Rat Cytokine/Chemokine Magnetic Bead Panel. The level of IL‐1α, IL‐1β, IL‐12p70, LIX, EGF, and VEGF increased significantly after 4 days of BMF smoke exposure (*p *= 0.018, 0.008, 0.043,0.001, 0.001 and 0.007, respectively). The level of IL‐1β, TNFα and LIX increased after 1 month (*p *= 0.038, 0.031 and 0.021, respectively). After 6 months of BMF smoke exposure, only the level of VEGF was higher than the control level (*p *= 0.014), indicating that high levels of inflammatory cytokines were induced in the early stage of exposure. Interestingly, levels of IL‐4 in BALF increased significantly after 4 days of exposure (*p *= 0.002) and returned nearly to control levels after 1 and 6 months of exposure (*p *= 0.124 and 0.118). The levels of IL‐1α, IL‐1β and IL‐12p70 were elevated in old control rats in our study, suggesting the inflammatory cytokines were associated with the age.

### 3.4 Phenotypic characterization of AMs polarization induced by BMF smoke exposure

**FIGURE 3:**
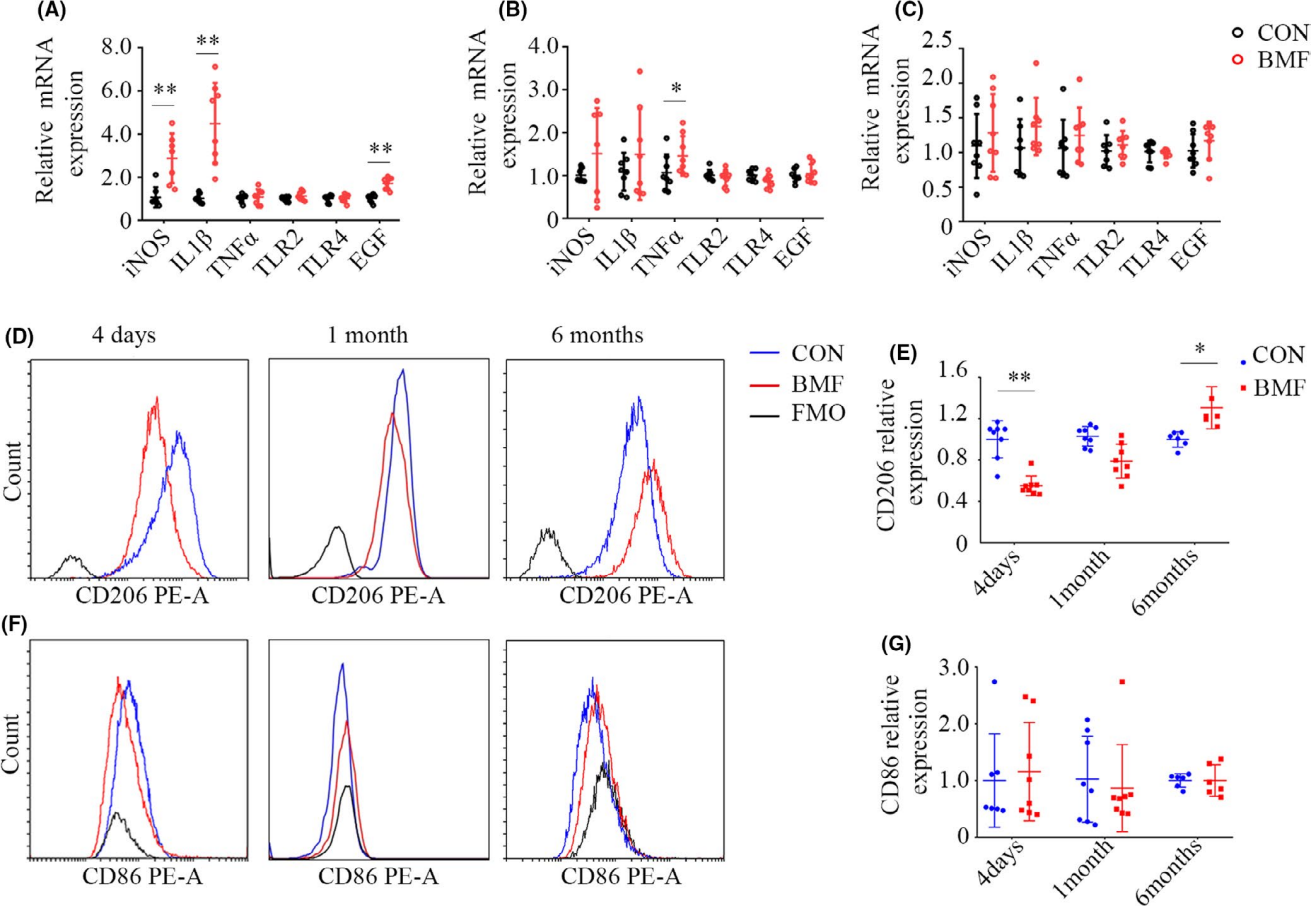
Smoke from BMF altered the expression of genes and surface markers in AMs. A: iNOS, IL‐1β and EGF mRNA expression upregulated in AMs exposed to BMF smoke for 4 days. B: TNFα mRNA expression in AMs had changed after 1 month of BMF exposure. C: iNOS, IL‐1β, TNFα, TLR‐2, TLR‐4 and EGF mRNA expression did not change after 6 months of exposure. D, E:Comparison of CD206 expression in AMs between BMF and CON groups. F, G:Comparison of CD86 expression in AMs between BMF and CON groups. Data represent the mean ±SD of a minimum number of six rats per group. *p < 0.05, ** *p* < 0.01, significantly different from CON groups. AMs, alveolar macrophages; BMF, biomass fuel smoke; CON, control; EGF, epidermal growth factor

We used qPCR to determine the mRNA expression of a few key genes of AMs following exposure to BMF smoke (Figure 3). The result showed that nitric oxide synthase (iNOS) and IL‐1β significantly ascended at 4 days of BMF smoke exposure (Figure 3A, *p* = 0.005 and 0.001), and the expression normalized after that time point. The expression of TNFα moderately elevated after 1 month of exposure (Figure 3B, *p* = 0.028), and declined after 6 months of exposure (Figure 3C, *p* = 0.374), consistent with levels of cytokines in BALF. The expression of toll‐like receptor (TLR) 2 and 4 did not vary over the course of the exposure, consistent with previous reports.^29^, ^30^ The level of EGF mRNA was upregulated in AMs of rats exposed for 4 days (Figure 3A, *p* < 0.01), consistent with the level of EGF protein in BALF.

To further assess the effect of BMF exposure on the dynamic phenotype change of AMs in rats, we also measured CD206 (M2 marker) and CD86 (M1 marker) in AMs (Figure 3). CD206 MFI decreased after 4 days of exposure (Figure 3D,E, *p* < 0.01), and increased nearly to control level after 1 month of exposure (*p *= 0.207), and was significantly higher than that of the control group after 6 months (*p *= 0.035). Conversely, CD86 MFI did no change in AMs during the exposure period (Figure 3F,G, *p* = 0.730, 0.831 and 0.995, respectively). Overall, the exposure initially reduced the expression of M2 marker in AMs, but the expression then increased with the duration of exposure.

### 3.5 BMF smoke exposure triggered signalling pathways of macrophage polarization and activation

**FIGURE 4:**
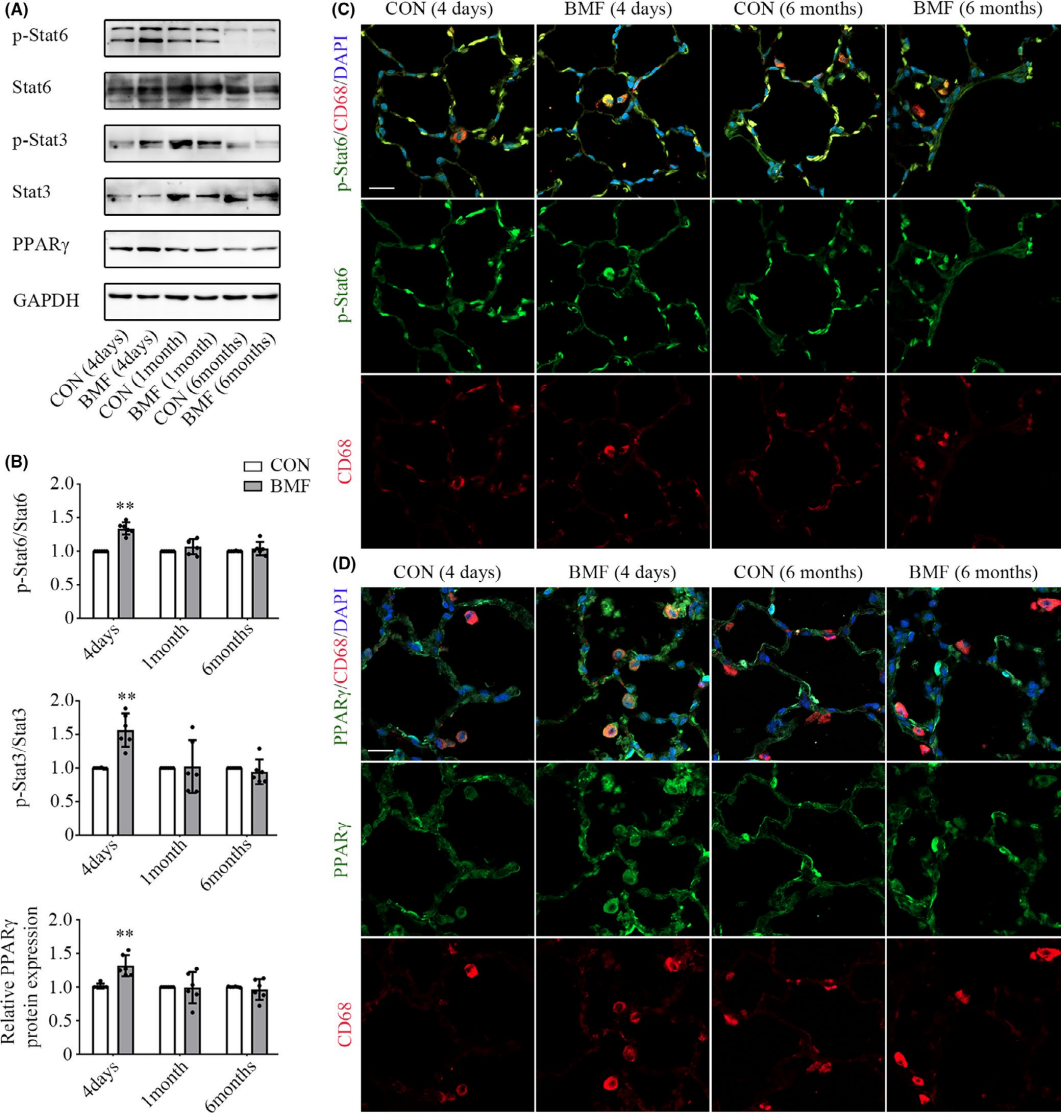
Smoke from BMF exposure triggered Stat6, Stat3 and PPARγ activation. A, B: Comparison of p‐Stat6, p‐Stat3 and PPARγ protein expression in lung tissue between groups. C: Time‐dependent activation of p‐Stat6 was examined by double immunofluorescence staining of p‐Stat6 (green) and CD68 (red). D: Time‐dependent activation of PPARγ was examined by double Immunofluorescence staining of PPARγ (green) and CD68 (red). Scale bar: 20 μm. The values in A and B represent the mean ± SD of a minimum number of six rats per group. *p < 0.05, **p < 0.01, significantly different from CON groups. BMF, biomass fuel smoke; CON, control; PPARγ, proliferator‐activated receptor γ

**FIGURE 5:**
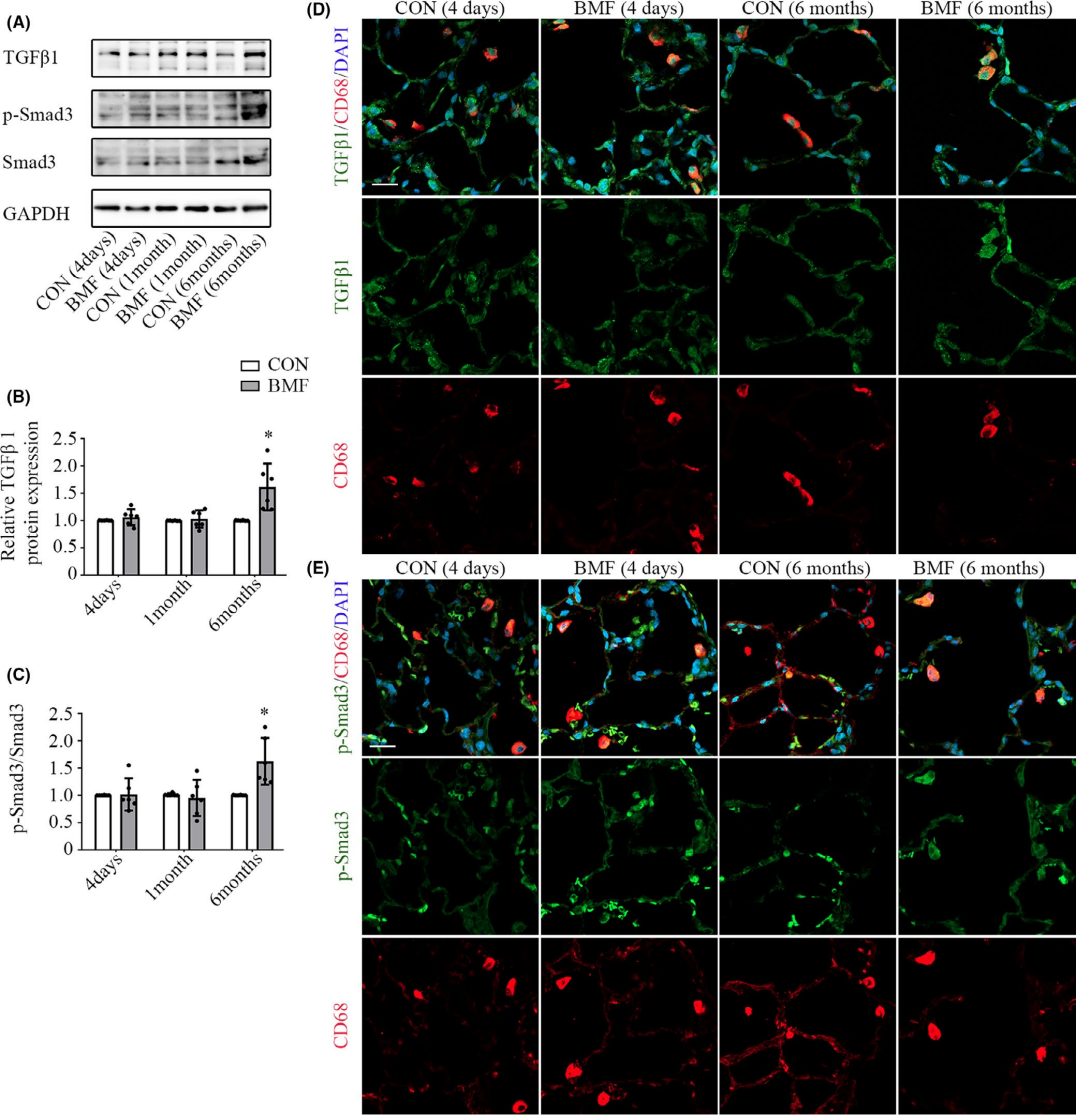
Smoke from BMF exposure triggered TGF‐β1 pathway activation. A, B: Western blotting of TGF‐β1 protein expression in lung tissue. A, C: Western blotting of protein levels of p‐Smad3 and Smad3 in lung tissue. D: TGF‐β1 in AMs was examined by double Immunofluorescence staining of TGF‐β1 (green) and CD68 (red). E: p‐Smad3 in AMs was also examined by double immunofluorescence staining of p‐Smad3 (green) and CD68 (red). Scale bar: 20μm. The values in B and C represent the mean ± SD of a minimum number of six rats per group. *p < 0.05, **p < 0.01, significantly different from CON groups

We sought to identify the signalling pathways involved in polarization and activation of AMs underexposure, especially those involved in M2 polarization to attenuate the inflammatory response and promote tissue remodeling.^13^, ^31^ We used qPCR, Western blotting and immunofluorescence to determine the mRNA and protein levels of Stat6, Stat3, PPARγ and TGF‐β1 in exposed rats. We also examined the phosphorylation of Stat6, Stat3 and Smad3. It showed that Stat6 mRNA expression in AMs increased significantly after 4 days of exposure (Figure S1A, *p *< 0.01) and descended to near‐control levels subsequently (Figure S1B,C, *p *= 0.149 and 0.661). Levels of p‐Stat6 increased after 4 days of exposure (Figure 4A,B, *p* < 0.01). Stat3 mRNA expression in AMs did not change at all during the exposure period (Figure S1A–C, *p *= 0.112, 0.209 and 0.832). In contrast, the level of p‐Stat3 elevated after 4 days of BMF exposure (Figure 4A,B, *p* = 0.003), and then declined to near‐control level after 1 and 6 months of exposure (Figure 4A,B, *p* = 0.898 and 0.484). PPARγ mRNA expression in AMs was upregulated after 4 days of exposure (Figure S1A, *p *< 0.01) and declined to normal level after that (Figure S1B,C, *p *= 0.66 and 0.543). PPARγ protein level in lung tissue did not differ between controls and exposure groups after 1 and 6 months of BMF exposure (Figure 4A,B, *p* = 0.934 and 0.572), but PPARγ protein level increased after 4 days of exposure (Figure 4A,B, *p* = 0.005), consistent with PPARγ mRNA expression in AMs. In contrast, TGF‐β1 and p‐Smad3 protein levels in lung tissue did not differ between controls and exposure groups after 4 days and 1 month of BMF exposure and increased after 6 months of exposure (Figure 5A–C, *p* = 0.017 and 0.017). Additionally, AMs expressing p‐STAT6, PPARγ, TGF‐β1 and p‐Smad3 were examined by immunofluorescence staining (Figures 4C,D and 5D,E). All four were induced in CD68‐positive cells (AMs) after 4 days and 6 months of BMF exposure, consistent with their protein levels in lung tissue.

### 3.6 PPARγ primed BMDMs exposed to PM into alternative macrophages

**FIGURE 6:**
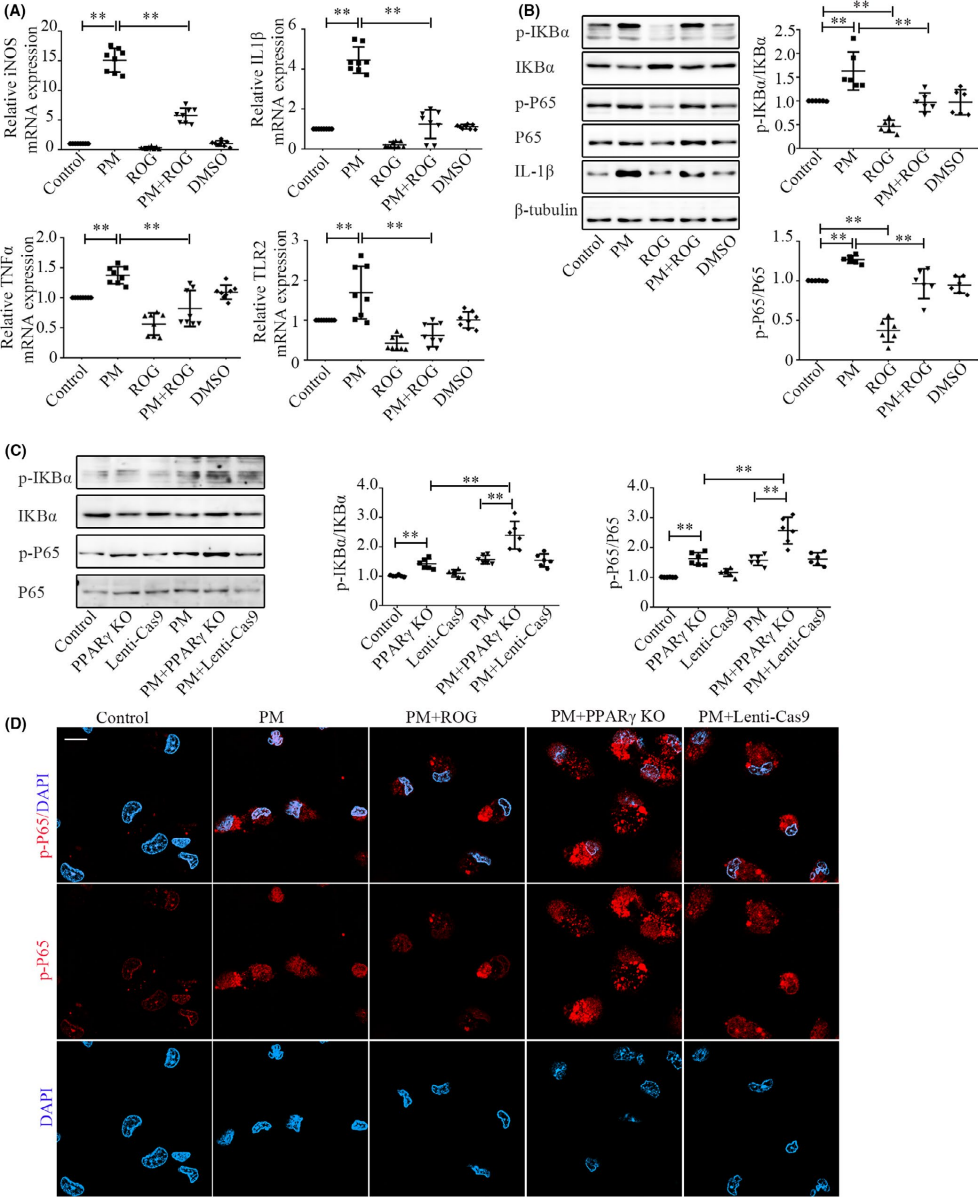
PPARγ reversed the M1 phenotype induced by biomass ambient particulate matter. A: Comparison of iNOS, IL‐1β, TNFα and TLR‐2 mRNA expression in BMDMs between groups after 24 h. B, C: Western blotting showed comparison of p‐IKBα expression in BMDMs between groups after 6 h, and comparison of p‐P65 and IL‐1βexpression after 48 h. D: Effect of PPARγ on nuclear translocation of p‐P65 in BMDMs. Nucleus translocation of p‐P65 in BMDMs between groups after 48 h was examined by immunofluorescence staining of p‐P65(red). Scale bar: 10 μm. The value in A–C represent the mean ± SD of a minimum number of six independent experiments. *p < 0.05, **p < 0.01, significantly different from control groups. BMDMs, bone marrow‐derived macrophages; PPARγ, proliferator‐activated receptor γ

To study whether PPARγ reversed the M1 phenotype induced by biomass ambient particulate matter and drove the macrophages into the M2 phenotype, we cultured BMDMs and stimulated them with PM extracted in our laboratory, then used the PPARγ agonist rosiglitazone and PPARγ knockout lentivirus as the intervention in vitro. The concentration of PM selected was 30 μg/ml PM (Figure S2A–C). The concentration of PPARγ agonist selected was 1.0 μM rosiglitazone (Figure S4A–C). The effect of PPARγ on inflammatory factors was determined via qPCR, Western blotting and immunofluorescence. PM‐induced BMDMs to express iNOS, IL‐1β, TNFα and TLR‐2 (Figure 6A, all *p *< 0.01), and BMDMs exhibited a pro‐inflammatory phenotype. Transcriptional factor PPARγ inhibited iNOS, IL‐1β, TNFα and TLR‐2 genes (Figure 6A, all *p *< 0.01). PM triggered phosphorylation of nuclear factor of kappa light polypeptide gene enhancer in B‐cell inhibitor (IKB) α from 6 to 12 h, and the levels of the p‐P65 began to increase after 6 h (Figure S3A,B). PPARγ overexpression significantly inhibited phosphorylation of P65 and IKBα (Figure 6B, *p* < 0.01 for all comparisons). PPARγ overexpression also inhibited the upregulation of IL‐1β protein level induced by PM (Figure 6B). Western blotting confirmed the loss of PPARγ in PPARγ knockout lentivirus cultures (Figure S5). PPARγ knockout lentivirus increased the levels of p‐P65 and p‐IKBα (Figure 6C, *p* < 0.01 for all comparisons). Immunofluorescence staining showed that PPARγ knockout lentivirus promoted p‐P65 nucleus translocation, while PPARγ overexpression inhibited p‐P65 nucleus translocation induced by PM (Figure 6D).

**FIGURE 7:**
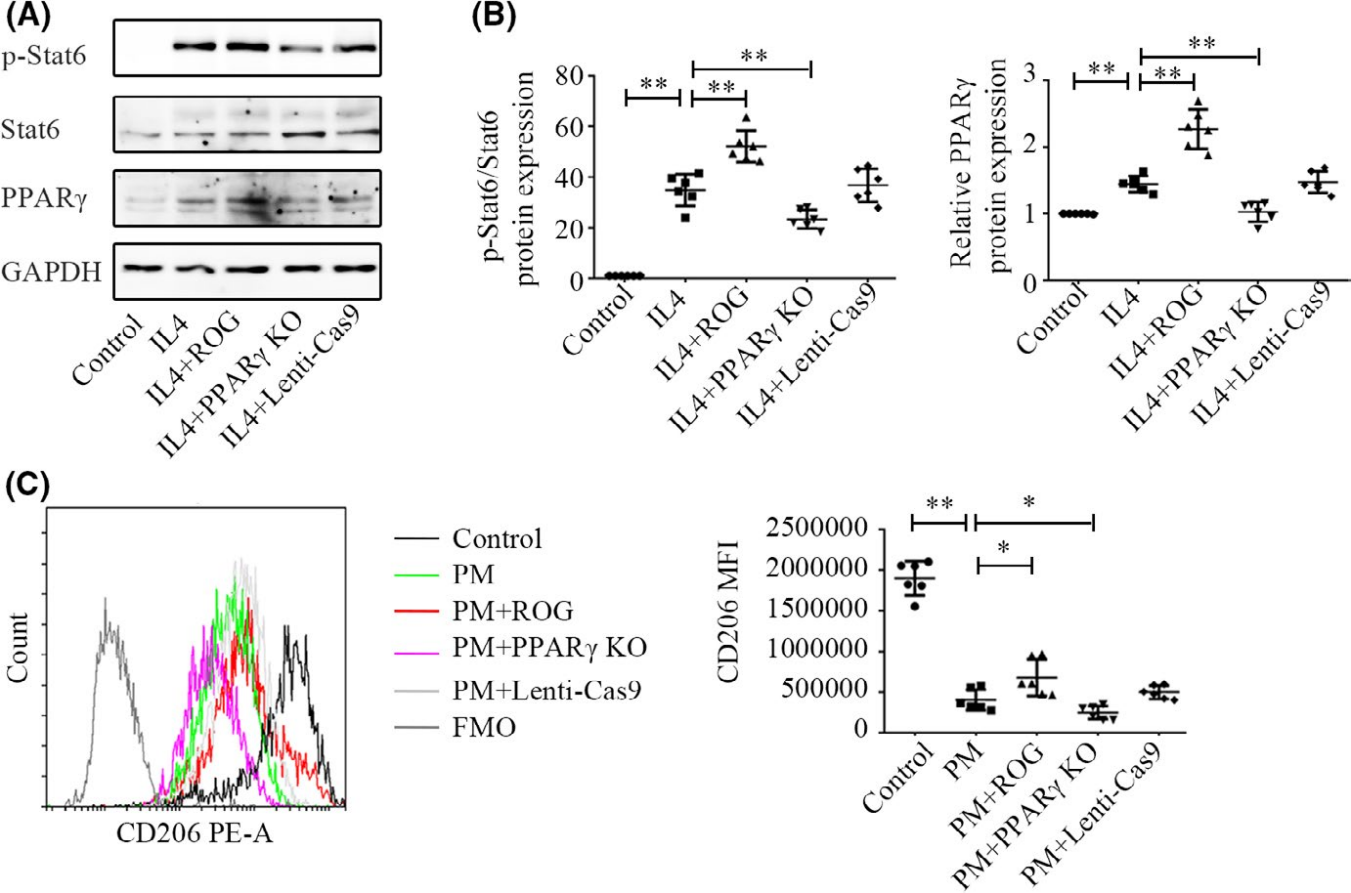
PPARγ primed BMDMs into the M2 phenotype. A, B:Western blotting of PPARγ and p‐STAT6 expression in BMDMs. C: Comparison of CD206 MFI in BMDMs between groups. The values in B and C represent the mean ± SD of six independent experiments. *p < 0.05, **p < 0.01, significantly different from control groups. BMDMs, bone marrow‐derived macrophages; PPARγ, proliferator‐activated receptor γ

In addition, we found that PPARγ in BMDMs was upregulated by stimulation with IL‐4 and promoted the expression of M2 markers. IL‐4 stimulated PPARγ and p‐STAT6 expression (Figure 7A,B, *p* < 0.01). PPARγ overexpression increased the level of p‐STAT6 (Figure 7A,B, *p* < 0.01), while PPARγ knockout lentivirus attenuated the phosphorylation of STAT6 (*p *< 0.01). Moreover, PPARγ overexpression upregulated CD206 expression (Figure 7C, *p* = 0.026), and PPARγ knockout lentivirus downregulated CD206 expression (Figure 7C, *p* = 0.041).

### 3.7 TGF‐β1 promoted CD206 expression in BMDMs

PM decreased CD206 expression (Figure S6, *p *< 0.01). TGF‐β1 attenuated the downregulation of CD206 induced by PM (Figure S6, *p* < 0.01), but had no effect on BMDMs that were not stimulated by PM (*p *= 0.522).

## 4 DISCUSSION

Indoor air pollution induced by biomass ambient particulate matter is strongly linked to the incidence and hospitalization rates of COPD. Our previous study showed that biomass ambient particulate matter retention in lung tissue‐induced pulmonary inflammation, airway remodelling and alveolar cavity enlargement.^32^, ^33^, ^34^ The chronic BMF smoke exposure model serves as a useful model to analyse how indoor air pollution promotes the progress of emphysema in lung. In this work, we demonstrated that the emergence of pro‐inflammatory macrophages eventually conversed into anti‐inflammatory macrophages following exposure to smoke from BMF, which initiated this plasticity in AMs. We also identified the signalling pathways regulating this functional conversion of AMs and the dynamic molecular changes that drove AMs into an anti‐inflammatory phenotype.

Airway inflammation, airway remodelling and alveolar cavity enlargement were observed in our BMF smoke exposure model. The early stage was characterized by airway inflammation, and the later stage was characterized by airway remodelling and alveolar cavity enlargement, in agreement with published observations in COPD models.^32^ Our previous study also showed that peak expiratory flow (PEF) and forced expiratory volume in 20 milliseconds/forced vital capacity (FEV_20_/FVC) decreased following a chronic exposure to BMF smoke, indicating lung dysfunction.^32^ CD68 is a specific macrophage marker in rats, mice, and humans, and F4/80 is expressed in mature macrophages in mice. CD11b is expressed in both rats and mice, but its expression is low in AMs.^35^ In the present study, we used CD68 as a marker of AMs and found that BMF exposure‐induced macrophages infiltration into the lungs, which increased with the accumulation of exposure time.

Surprisingly, molecular analyses revealed that the emerging pro‐inflammatory macrophages shared a feature of the anti‐inflammatory phenotype, which partially overlapped but was also distinct, including the co‐expression of mRNA encoding of IL‐1β, iNOS and PPARγ. The result also indicated that AMs produced pro‐inflammatory factors to damage lung tissue and then skewed towards an anti‐inflammatory phenotype after short‐term exposure. After 6 months of BMF smoke exposure, the protein level of TGF‐β1 increased, and airway and lung tissue were remodelled, resulting in the COPD pathology. The whole course from pro‐inflammatory phenotype to anti‐inflammatory phenotype, and then to the chronic pathology, indicated an interaction between BMF exposure and the pulmonary immune system.

Inflammation occurred in the early stage of BMF smoke exposure. Camila Oliveira da Silva and co‐workers observed a dynamic change in cytokine production in mice exposed to cigarette smoke (CS),^36^ involving an initial increase in TNFα and NO and a subsequent decline, although a 30 days of CS exposure increased TGF‐β1 production in the lung.^36^ We also found that the levels of IL‐1α, IL‐1β, IL‐12p70, LIX as well as IL‐4 in BALF increased after a short‐term exposure to BMF smoke. IL‐1α and IL‐1β, which are mainly produced by activated monocytes and macrophages, enhance B‐cell proliferation and maturation, natural killer cells (NK) cytotoxicity, pro‐inflammatory chemokine expression and acute‐phase protein expression. IL‐12p70, produced by monocytes and macrophages, can further act on lymphocytes and effectively promote Th1 response in COPD. LIX, which is a small cytokine of the CXC chemokine family, is produced by epithelial cells following stimulation with IL‐1 or TNFα and promotes the chemotaxis of neutrophils. Dynamic changes of pro‐inflammatory cytokines revealed that the most severe inflammatory injury is in the early stage of exposure. Interestingly, levels of the Th2 cytokine IL‐4 increased simultaneously to promote AMs towards the anti‐inflammatory phenotype. Levels of pro‐inflammatory cytokines gradually decreased over the course of the exposure. Upregulation of IL‐4 and the attenuation of inflammation were chronologically sequenced in COPD models, as seen previously.^37^ Studies have reported that M2 can secrete EGF and VEGF to promote tissue repair,^17^, ^38^ suggesting that both factors are upregulated under the action of Th2 cytokine in the early stage of exposure. Shaykhiev and co‐workers provided transcriptome‐based evidence that smoking induces reprogramming towards M2 polarized macrophages in COPD patients, suggesting that AMs were likely involved in the pathogenesis of COPD in a non‐inflammatory manner.^39^ However, the clinical study did not track immune changes over time. Our study provided data on dynamic phenotype and functional changes in AMs during exposure to BMF smoke.

Macrophage polarization is controlled by signal pathway molecules, but the identity of these signal pathways is unclear in COPD. In the present study, the anti‐inflammatory phenotype was most prevalent, although it overlapped with pro‐inflammatory phenotype after 4 days of exposure. We therefore focused more on signal pathways regulating the anti‐inflammatory phenotype. IL‐4 skews macrophages towards the M2 phenotype and activates Stat6 and Stat3. Stat6 modulates multiple genes associated with the M2 phenotype, including CD206, arginase 1 and resistin‐like α.^40^ We found phosphorylation of Stat6 and Stat3 increased after 4 days of exposure, consistent with upregulation of IL‐4. This supports the hypothesis that Stat6 and Stat3 signals participate in activation of M2 in the early stage of exposure.

PPARγ is an important transcription factors of M2 and primes monocytes into alternative activated macrophages.^20^, ^41^ The dynamic change of PPARγ has not been reported previously. We found that PPARγ elevated during the most severe inflammation periods, and then returned to the control level as the inflammation subsided. We also demonstrated that PPARγ was upregulated in BMDMs by IL‐4 in vitro. The Th2 cytokine IL‐4 is required for the development of the M2 phenotype, and IL‐4‐mediated signals stimulate PPARγ expression.^20^, ^42^, ^43^ In addition, our study showed that PPARγ inhibited the activation and nuclear translocation of NFκB p65 via by inhibiting the phosphorylation of IKBα, and upregulated the expression of the M2 markers CD206 and p‐STAT6, suggesting that PPARγ reversed M1 phenotype induced by PM, and drove the macrophages into the M2 phenotype rapidly in a protective manner. Drugs regulating PPARγ provide a potential interventional target in COPD; rosiglitazone was reported to inhibit cigarette smoke‐induced pulmonary inflammation^44^ and reduce exacerbations by attenuating pulmonary inflammation and decreasing bacterial burdens,^45^ suggesting PPARγ may therefore be an effective approach in treating COPD.

TGF‐β1 is another important regulatory molecule of M2, and critical for the development and maturation of AMs. TGF‐β1 can also inhibit the expression of macrophage‐derived inflammatory genes.^46^, ^47^ M2 participates in the development of fibrosis and contributes to disease pathogenesis. M2‐derived TGF‐β1 promotes tissue remodelling and wound repair by blocking the degradation of the extracellular matrix's degradation and eliciting synthesis of interstitial fibrillar collagens.^13^ Besides, airway remodelling via the TGF‐β1 pathway has been shown to lead to the thickening of the small‐airway wall. TGF‐β1 in AMs may be involved in the mechanism of airway remodelling. Our study showed that AMs maintained an anti‐inflammatory phenotype characterized by elevated CD206 and TGF‐β1 markers without stimulation of Th2 cytokines after 6 months of exposure. We also found that TGF‐β1 promoted CD206 expression in BMDMs in vitro. Chronic exposure to BMF smoke‐induced TGF‐β1 production and activated the downstream signal Smad3, which is involved in tissue remodelling. Activation of TGF‐β1 in the late stage of exposure indicated the role of TGF‐β1 in regulating M2 polarization and participating in lung tissue remodelling.

In summary, our study describes the dynamic phenotype and functional changes of AMs during exposure to BMF smoke, suggesting a pro‐inflammatory role of AMs in the early stage of COPD and an anti‐inflammatory role associated with tissue remodelling in the latter stage. Identification of more signal pathway molecules involved in AMs polarization may provide potential targets for the COPD treatments.

## CONFLICTS OF INTEREST

The authors declare that no conflict of interest exists.

## AUTHOR CONTRIBUTIONS

**Shenlin Wang:** Conceptualization (lead); Data curation (lead); Formal analysis (lead); Funding acquisition (supporting); Methodology (lead); Project administration (lead); Supervision (lead); Validation (lead); Visualization (lead); Writing – original draft (lead); Writing – review & editing (lead). **Yuhua Chen:** Data curation (equal); Formal analysis (equal); Methodology (equal); Project administration (equal); Validation (equal). **Wei Hong:** Conceptualization (equal); Methodology (equal); Project administration (equal); Resources (equal); Visualization (equal). **Bing Li:** Conceptualization (equal); Data curation (equal); Methodology (equal); Project administration (equal); Resources (equal); Validation (equal); Visualization (equal). **Yumin Zhou:** Funding acquisition (equal); Methodology (equal); Project administration (equal); Resources (equal); Supervision (equal); Validation (equal). **Pixin Ran:** Conceptualization (lead); Funding acquisition (lead); Methodology (equal); Project administration (lead); Resources (lead); Supervision (lead); Validation (lead); Visualization (lead).

## Supporting information

Supplementary Material

## Data Availability

The data that support the findings of this study are openly available.
