# Supplementary material for: Chronic exposure to biomass ambient particulate matter triggers alveolar macrophage polarization and activation in the rat lung
Source: J Cell Mol Med. 2022 Jan 6;26(4):1156–68. doi: 10.1111/jcmm.17169 (PMC8831949; doi:10.1111/jcmm.17169)
Supplement: Supplementary file 1 — Supplementary Material [file JCMM-26-1156-s001.docx]

**Chronic exposure to biomass ambient particulate matter triggers alveolar macrophage polarization and activation in the rat lung**

Shenlin Wang^1,3^, Yuhua Chen^2^, Wei Hong^2^, Bing Li ^2^, Yumin Zhou^1^, Pixin Ran^1*^

^1^State Key Laboratory of Respiratory Diseases, National Clinical Research Center for Respiratory Diseases, Guangzhou Institute of Respiratory Health, The First Affiliated Hospital of Guangzhou Medical University, Guangzhou, Guangdong, China

^2^GMU-GIBH Joint School of Life Sciences, Guangzhou Medical University, Guangzhou, Guangdong, China

^3^Department of Respiratory Medicine, Ningxia Hui Autonomous Region People's Hospital, The First Affiliated Hospital of Northwest University for Nationalities, Yinchuan, Ningxia, China

^*^Correspondence author. E-mail: pxran@gzhmu.edu.cn

**Supplementary Table 1** Primer sequences

| Gene | Forward | Reverse |
| --- | --- | --- |
| iNOS | 5’-TCCTCAGGCTTGGGTCTTGT-3’ | 5’-GTGAGGAACTGGGGGAAACC-3’ |
| IL-1β | 5’-AGGCTGACAGACCCCAAAAG-3’ | 5’-CTCCACGGGCAAGACATAGG-3’ |
| TNFα | 5’-AACTCGAGTTACAAGCCCGTAG-3’ | 5’-GTACCACCAGTTGGTTGTCTTTG-3’ |
| EGF | 5’-TTGAATCCGTGGACCGCTAC-3’ | 5’-CACAAACCAAGGTTGGGGAC-3’ |
| TLR-2 | 5’-ACTCAAGAGCATCGGCTGG-3’ | 5’-CAATGTCAGAACCCGGAGGT-3’ |
| TLR-4 | 5’-CGCTTTCAGCTTTGCCTTC-3’ | 5’-CTCCAGAAGATGTGCCTCCC-3’ |
| Stat6 | 5’-CCCCAGAAAAACTGCAACGG-3’ | 5’-TCCTGGTCTCCCTTACTCGG-3’ |
| Stat3 | 5’-ATCCTAAGCACAAAGCCCCC-3’ | 5’-CTGGGTCAGCTTCAGGGTG-3’ |
| PPARγ | 5’-GGAGATCCTCCTGTTGACCC-3’ | 5’-TGGGTCAGCTCTTGTGAACG-3’ |
| TGF-β1 | 5’-AGCCCTGTATTCCGTCTCCT-3’ | 5’-ATTCCTGGCGTTACCTTGG-3’ |
| GAPDH | 5’-AGTGCCAGCCTCGTCTCAT-3’ | 5’-GATGGTGATGGGTTTCCCGT-3’ |

**Supplementary Table 2** The concentration of PM, O_2_, CO, NO and SO_2_ level in the exposure chamber

|  | CON | BMF |
| --- | --- | --- |
| PM_1_ mass concentration(mg/m^3^) | 0.0046±0.0030 | 27.77±8.66 |
| PM_2.5_ mass concentration(mg/m^3^) | 0.0050±0.0027 | 28.07±8.84 |
| PM_10_ mass concentration(mg/m3) | 0.0050±0.0026 | 28.23±8.86 |
| O_2_(%) | 20.99±0.036 | 20.23± 0.0014 |
| CO(ppm) | - | 55.16±13.77 |
| NO (ppm) | - | - |
| SO_2_ (ppm) | - | - |

Figure S1


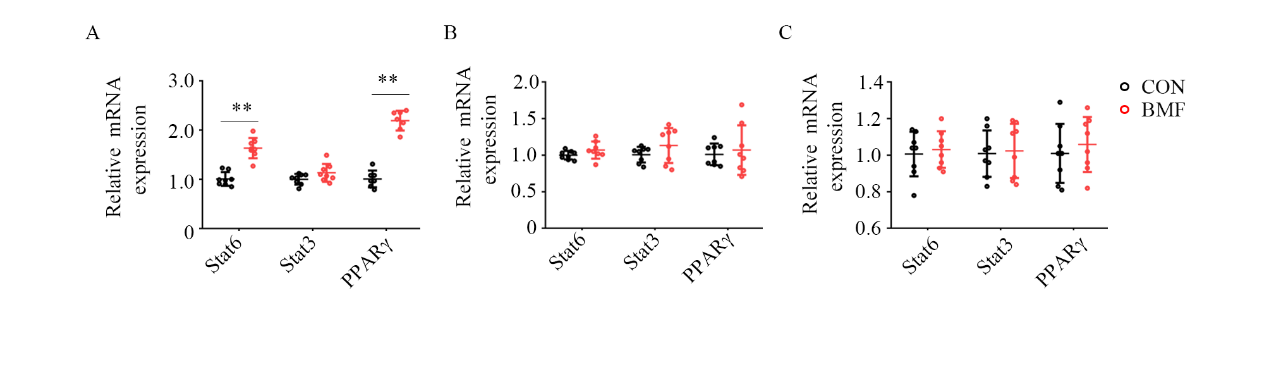


**Supplementary Figure 1. Smoke from BMF triggered Stat6, Stat3, and PPARγ mRNA expression.**

**A:** Comparison of Stat6, Stat3, and PPARγ mRNA expression in alveolar macrophages between groups after 4 days BMF exposure. **B:** Stat6, Stat3, and PPARγ mRNA expression in alveolar macrophages after 1 month of exposure. **C:** Stat6, Stat3, and PPARγ mRNA expression in alveolar macrophages after 6 months of exposure. The value in **A** and **B** represent mean ±SD of a minimum number of six rats per group. **p*<0.05, ** *p*<0.01, significantly different from CON groups.

Figure S2


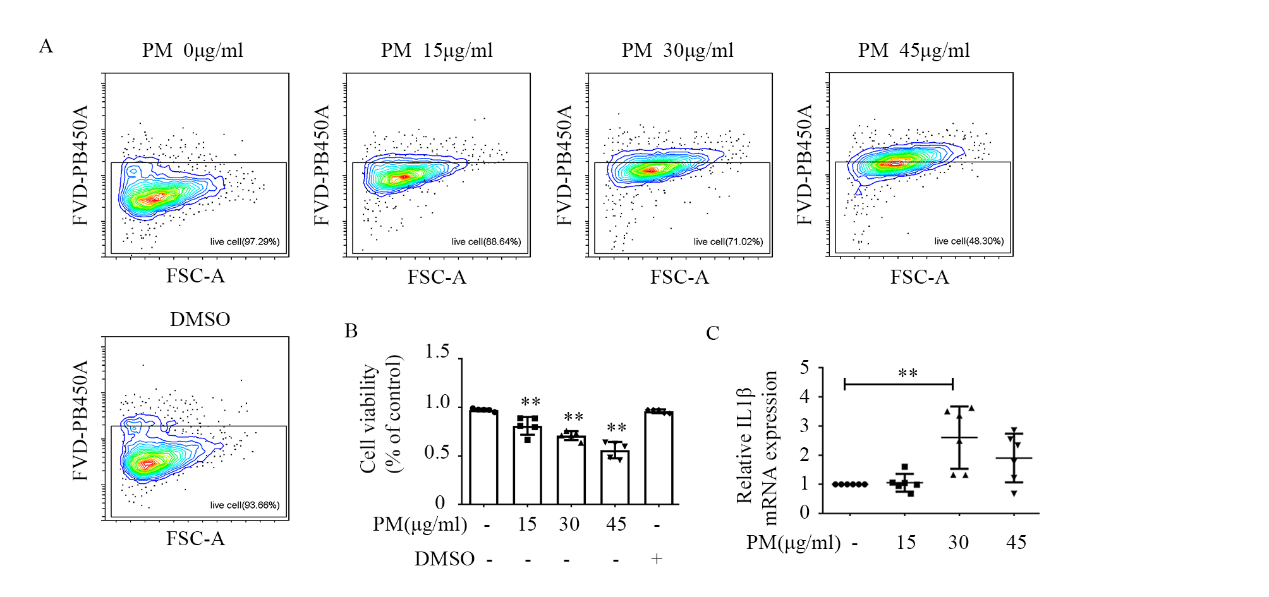


**Supplementary Figure 2. Effects of PM on cell viability and inflammation. A, B:** The toxicity of PM for BMDMs was assessed by Flow cytometry. The effect of PM on cell viability was dose-dependent. Cell survival was (80.95±9.10)%, (70.92±4.55)%, and (56.03±8.42)% at the concentration of 15μg/ml, 30μg/ml, and 45μg/ml respectively. **C:** Comparison of IL-1β mRNA expression in BMDMs at the concentration of 0 μg/ml, 15 μg/ml, 30 μg/ml, and 45 μg/ml. IL-1β mRNA expression elevated obviously at the concentration of 30μg/ml(*p*=0.004), however, IL-1β mRNA expression had no change compared to the control group at the concentration of 45 μg/ml(*p*=0.056). As a result, 30 μg/ml PM was selected as intervention concentration. The value in **B** represents mean ±SD of five independent experiments. The value in **C** represents mean ±SD of six independent experiments.. **p*<0.05, ** *p*<0.01, significantly different from untreated group.

Figure S3

**
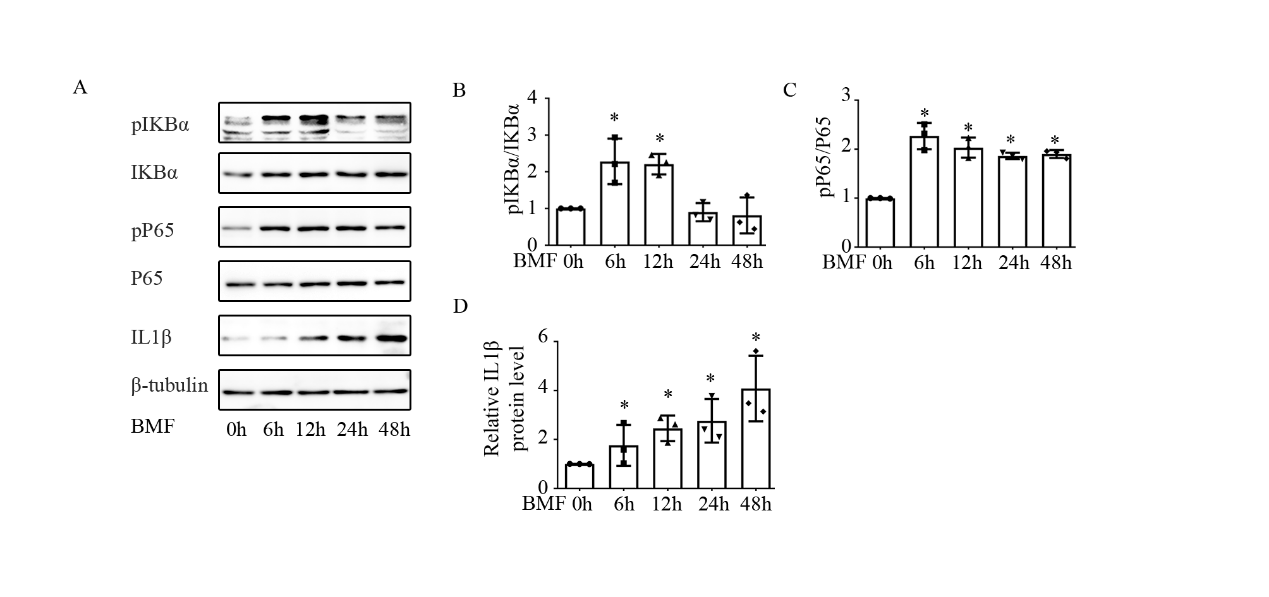
**

**Supplementary Figure 3. Effect of PM on the activation of NF-κB signaling pathway in BMDMs. A, B:** BMDMs were treated with 30 μg/ml PM, levels of phosphorylated IKBα were assessed by WB. Phosphorylated IKBα elevated from 6 h to 12 h. **A, C:** Levels of phosphorylated P65 were assessed by WB. After 6 h with PM stimulation, p-P65 began to rise. **A, D:** Levels of IL-1β were assessed by WB. IL-1β increased after 6 h, and reached the peak at 48 h. Data was represented mean ±SD of three independent experiments. **p*<0.05, ** *p*<0.01, significantly different from untreated group.

Figure S4


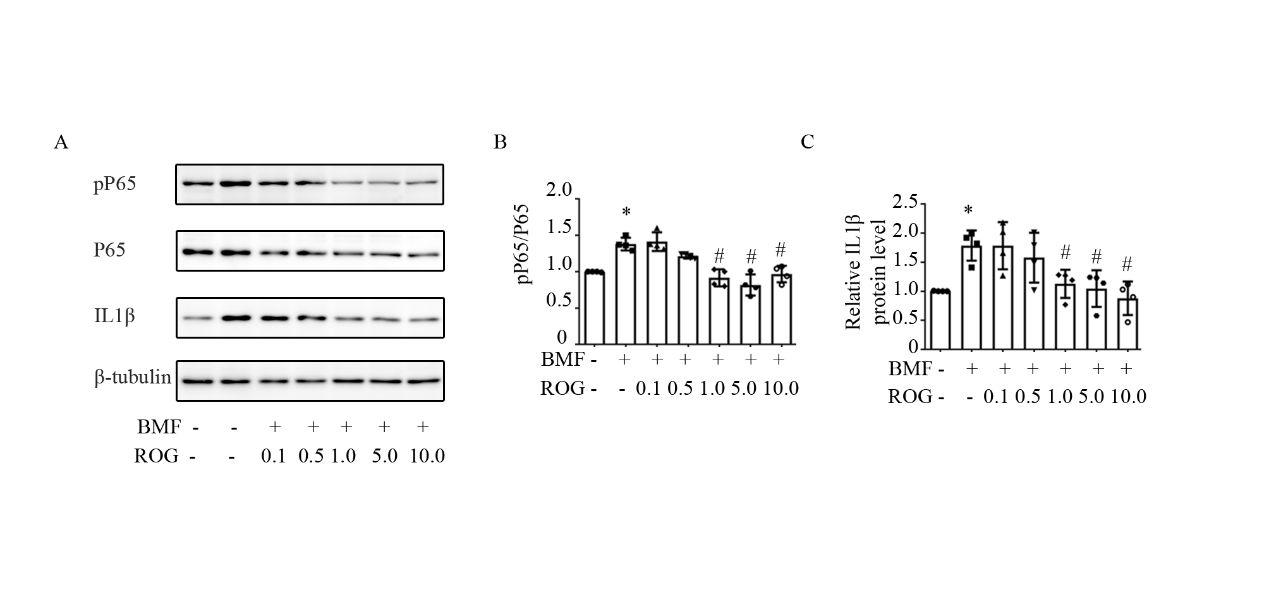


**Supplementary Figure 4. Effect of** **rosiglitazone on the activation of p-P65 and IL-1β in BMDMs.** BMDMs were treated with rosiglitazone (0.1, 0.5, 1.0 5.0, and 10.0 μm ) for 48 h. **A,B:**  Levels of phosphorylated P65 were assessed by WB. **A,C:** Levels of IL-1β were assessed. Data was represented mean ±SD of 4 independent experiments. **p*<0.05, significantly different from untreated groups. #*p*<0.05, significantly different from PM group.

Figure S5


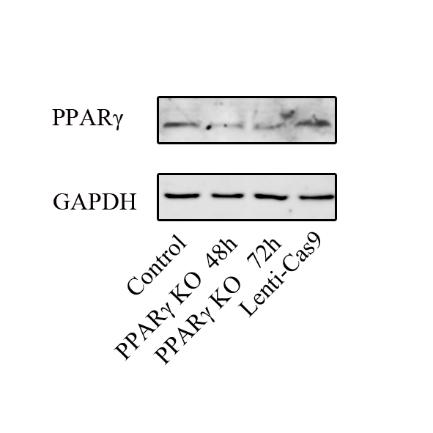


**Supplementary Figure 5. Effect of PPARγ KO lentivirus on PPARγ expression at 48 h and 72 h.**

Figure S6


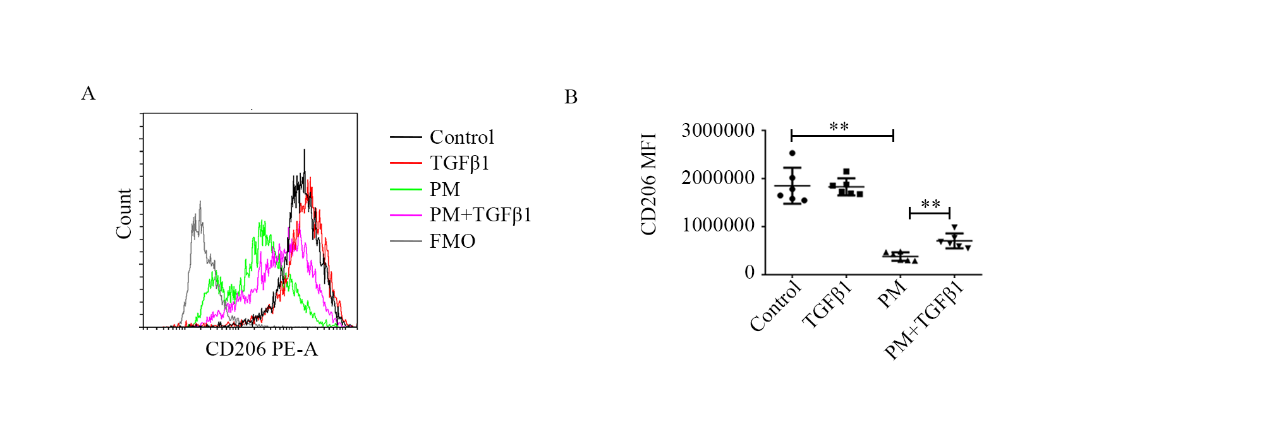


**Supplementary Figure 6. TGF-β1 promoted CD206 expression in BMDMs.** Comparison of CD206 MFI in BMDMs between groups. The value in B represents mean ±SD of six independent experiments. **p*<0.05, ** *p*<0.01, significantly different from control groups.
